# Mandated Parent Education: Applications, Impacts, and Future Directions

**DOI:** 10.1007/s10567-024-00488-1

**Published:** 2024-05-18

**Authors:** Cheri J. Shapiro, Crystal Hill-Chapman, Stephanie Williams

**Affiliations:** 1 Institute for Families in Society, College of Social Work, University of South Carolina, 1600 Hampton St., Suite 507, Columbia, SC 29208 USA; 2 Department of Psychology, Francis Marion University, Florence, SC USA

**Keywords:** Mandated, Parenting education, Court-ordered, Evidence-based interventions, Population-wide parenting

## Abstract

Mandated participation in parent education programs is a common practice across the United States for families who are undergoing divorce or who are involved in the child welfare or juvenile justice systems. Mandates to participate in parenting programs create substantial challenges for families, service providers, and service systems. Furthermore, the type and quality of the parenting services accessed vary widely, and their impacts need to be better understood. To address this need, an overview of the current state of the empirical literature on the impacts and outcomes of mandated parenting interventions for divorce and in child welfare and juvenile justice settings is provided, and suggestions to the field are offered to refine research related to mandated parenting programs. Given the challenges that mandated parenting programs pose, an alternative approach that views parenting through a public health lens is highlighted to build on the growing body of research on the impacts of population-wide applications of parenting support programs, and as a possible way to decrease the number of parents who are required to attend parenting programs. Opportunities to advance universal parenting support within a range of community settings, including primary care, early childhood education, and community mental health systems are offered. Gaps in knowledge regarding mechanisms of action of universal supports and impacts on the number of parents mandated to treatment are highlighted, and future directions for research in this area are suggested.

Mandated (court-ordered) parent education is a common practice across the United States for families who are undergoing divorce and for families who are involved in the child welfare and/or juvenile justice systems each year. Across these systems, this can translate to a large number of parents mandated to attend parenting programs each year. For example, the divorce rate in 2022 was 14.56 per 1000 married women, representing 989,518 women who reported divorcing in the past year (Loo, 2023) and the majority of states require completion of a parenting program as a condition of divorce. Within the child welfare system, mandates to attend parenting programs are typically made as a result of child maltreatment. According to the Centers for Disease Control and Prevention, child maltreatment is estimated to impact one in seven children (Fast Facts, 2023), with 600,000 children identified as victims of child maltreatment in 2021 (National Child Abuse Statistics from NCA, n.d.). Within the criminal justice system, juvenile courts managed 722,600 delinquency cases in 2019 (Puzzanchera et al., 2022). Widespread use of mandated parenting programs in these systems poses substantial challenges for families, service providers, and service systems and may not result in the desired outcomes, such as reductions in parent conflicts during divorce, reductions in child maltreatment, or reductions in rates of juvenile delinquency. To address these concerns, we examine the history of mandated parenting programs, detail challenges to the implementation of mandated parenting programs, and provide an overview of the impacts of mandated parenting programs across key applications (i.e., child maltreatment, juvenile delinquency). We then discuss alternative approaches, focusing on existing research that involves viewing parenting through a public health lens and that promotes universal access to high-quality, evidence-based parenting supports. We highlight how implementing high-quality, population-level parenting supports through existing service systems that touch many parents (e.g., early childhood education and primary care) holds promise to reduce the use of mandated parenting interventions and the inherent challenges that mandated participation poses. We close with consideration of gaps in our knowledge of the impacts of universal parenting programs and provide suggestions for future research on mandated parenting programs.

## Origins of Mandated Parenting Programs

Parents have long been seen as both contributors to child and adolescent behavioral challenges and key figures in treatment approaches to address these challenges. The role of parents as interventionists in addressing child and adolescent problem behaviors was established in the 1960s and 1970s with the early work of Patterson and colleagues (e.g., Margolin & Patterson, 1975; Patterson & Fagot, 1967). These early studies focused on the social environmental influences (i.e., family influences) on child behavior. This led to a growing body of research establishing the effectiveness of parents as change agents for their children and increasing focus on parent training as an intervention strategy (e.g., Eyberg & Matarazzo, 1980; Moore & Patterson, 2009; Webster-Stratton, 1985; Wells et al., 1980). As this body of research on parenting interventions developed and grew, court systems, with supporting legislation, began to implement or require parenting programs in both the US and the UK (Burney & Gelsthorpe, 2008). In the U.S., for example, in a survey of 191 juvenile court judges, 21% indicated group education or therapy for parents of delinquent youth (Windell & Windell, 1977). Within this group of judicial respondents, 15% noted that parents were mandated to attend by court order, and 22% used a mix of voluntary and involuntary participation (Windell & Windell, 1977, p. 461).

Further tracing the origins of mandated parenting interventions, it is likely that the publication of a seminal paper entitled “The Battered Child Syndrome” by Henry Kempe and colleagues (Kempe et al., 1962) helped to spur this development. Kempe et al. (1962) served as a call to action for medical professionals to identify children who were victims of severe physical abuse, and it was further suggested that “psychiatric factors” in parents were the leading cause of this then-novel syndrome (Kempe et al., 1962). Consequently, mandates to identify and report suspected maltreatment were codified by the passage of the U.S. Child Abuse Prevention and Treatment Act (CAPTA) in 1974. This federal legislation included funding for states that had established mandated systems of reporting and investigating potential cases of child abuse and neglect. As parents were and are noted as the primary perpetrators of child maltreatment (USDHHS, 2024), it is unsurprising that parents continue to be the targets of mandated parenting interventions to address child maltreatment, especially given the goals of child welfare to keep children at home or to reunify families (Barth et al., 2005).

## Expansion of Mandated Parenting Interventions for Divorce

Beyond the use of mandated parenting education to address juvenile delinquency and child maltreatment, in the 1970s, family courts began to use mandated mediation to manage cases involving divorce and/or custody disputes. Family court judges in California were the first to allow the discretion of mandating parent participation in mediation to resolve custody disputes in 1980; by 1995, this practice had expanded to most states (Press, 2013, p. 49). By 2001, parent education courses designed to prepare parents to manage parenting conflicts in the context of divorce and were offered statewide in 28 states (Press, 2013, p. 52). Research on the impacts of mandated parent education for divorcing parents grew (e.g., Kramer & Washo, 1993; McKenry et al., 1999; Petersen & Steinman, 1994; Roeder-Esser, 1994). A review of these programs published in 2008 reveals that these mandatory parenting programs for divorce were codified into law in all but a handful of states (Pollet & Lombreglia, 2008).

Despite the widespread use and popularity of these programs in the family court system, the programs used vary widely in content, focus, and goals. To address this variety of programs, Salem and colleagues (2013) conceptualized divorce as a public health problem, proposing a public health model to better organize parenting programs for divorce offered through family courts. In this model, mandated parenting programs are at the highest (indicated) intervention tier (Salem et al., 2013). Despite the promise of this conceptual framework that offers a pathway to reduce the number of parents mandated to parenting programs, there does not appear to be evidence of uptake in family court systems. Consistent with trends in the use of technology for the delivery of parenting programs (David et al., 2024), current research on mandated parenting programs for divorce focuses on the use of technology and evaluations of online divorce education for parents (Bowers et al., 2011, 2014; Tomlinson et al., 2021; Yamaguchi & Randel, 2021). Importantly, despite mandates to attend these programs, pre-post studies and randomized trials have documented improvements in outcomes including parenting, interparent conflict, and child behavior (Wolchik et al., 2022).

In sum, the origins of mandated parenting programs are rooted in research demonstrating the impacts of parents as agents of change to address child behavior concerns. These programs grew to become a common requirement via family courts for parents involved in divorce, child welfare, and juvenile justice systems. Despite the continued popularity of mandated parenting programs and their growth over time, these programs pose specific challenges for parents, providers, and service systems.

## Challenges of Mandated Parenting Programs for Parents and Providers

Mandating participation in parenting programs presents challenges for individuals required to attend these programs, as well as for program providers. The most obvious challenge for parents is the requirement to participate in a parenting program. Required attendance involves practical considerations, including skills to self-organize tasks to facilitate attendance, which could involve altering work hours, finding child care, obtaining transportation, and overcoming perceptual barriers to participation, such as perceived stigma, as noted by parents (e.g., Mytton et al., 2014; Pinto et al., 2024). Mandating program attendance does not change these barriers that are most likely to negatively impact parents with the greatest levels of need, including those living in rural areas or those living in poverty.

A second more substantial concern for mandated parenting programs is that attendance (often used to assess program engagement) and participation are not identical constructs (Becker et al., 2015). Beyond attendance, there are challenges with parent/family engagement in treatment (and with how engagement is assessed, measured, and addressed; see Becker & Chorpita, 2023). Challenges to engagement are unlikely to be mitigated and are, in fact, are likely to be exacerbated by mandates to attend parenting programs. Mandates to attend serves as an external driver and can impact parent motivation, participation in services, and compliance with intervention tasks such as homework to practice skills. This attendance vs. participation dilemma for parents mandated to services represents an important challenge for providers that has long been recognized (Dinkmeyer, 1999). For meaningful changes to occur in parenting, engagement with and active participation in the services provided are needed. Indeed, even when services are not mandated, stigmatization or guilt may serve as barriers to engagement (Mytton et al., 2014). Furthermore, as noted by Hock and colleagues in a systematic review of qualitative studies, parent-reported barriers to engagement include factors at the parent, provider, and intervention levels of the social ecology (Hock et al., 2015). These include behavioral barriers such as readiness to change, participant constraints such as stigma and competing demands, and social/cultural barriers such as low income or low literacy (Hock et al., 2015).

As well as posing challenges for parents, mandated parenting programs present substantial challenges for service providers. One of the most important challenges is overcoming the adversarial nature of the relationship within mandated services in general (Sotero et al., 2017) and when services within the child welfare or juvenile justice systems are mandated (Burke et al., 2014). Establishing a working alliance, a bidirectional construct, is important for treatment and appears to require more effort from the provider when services are mandated, at least initially (Sotero et al., 2017). Indeed, in a multi-level meta-analysis of family-involved interventions for youth behavior, the alliance’s quality was positively associated with intervention outcomes (Welmers-van de Poll et al., 2018). Complicating the formation of a therapeutic alliance in mandated treatment is the element of therapist control; in examining perceptions of affiliation and control among adult clients, mandated services were found to involve a higher degree of control (Manchak et al., 2014). Thus, therapists and others working with individuals mandated to intervention must be prepared for the effort and skill required to balance the mandated context of the service and inherent control with the ability to form an effective relationship.

Another possible impact of mandated services for providers and parents is the effect on the broader construct of parent self-regulation. Promoting parent self-regulatory capacity is a core goal of parenting interventions (Sanders, 2008; Sanders & Mazzucchelli, 2013) for two important reasons. First, parental ability to regulate their emotions and stress responses is critical to building emotion regulation skills in their children (Havighurst & Kehoe, 2017; Morris et al., 2017). Second, emotion regulation difficulties have been conceptualized as central to multiple types of psychopathology, including anxiety and depression in adults (Sloan et al., 2017), that can negatively impact parenting and child development (Goodman et al., 2011). Parental self-regulation is a complex construct that includes identifying when changes in parenting approaches are needed and becoming confident and competent to identify and solve parenting challenges (Sanders & Mazzucchelli, 2013). External requirements for attendance at parenting interventions have the potential to alter parent motivations and attributions for participation (“because I have to”), which, along with challenges in establishing a strong therapeutic alliance in mandated services, can limit the level of self-reflection required to produce meaningful or lasting changes in parenting behaviors. Thus, mandating intervention can undermine, at the start, a powerful source of motivation to change. Engaging in mandated services can also impact parent identity; for example, a qualitative study of 35 parents revealed positive and negative impacts on parent identity (Wolford & McWey, 2020). When coupled with limited engagement in parenting support and intervention services (Canfield et al., 2023), even when services are home-based (Guastaferro et al., 2020), mandates to attend parenting programs create barriers to harnessing parent self-regulatory processes which may be part of the very conditions that ultimately contribute to mandates to attend parent education.

## Challenges for Service Systems

In addition to the challenges mandated parenting programs pose for parents and providers, such mandates also present challenges for service systems providing these interventions. Parents involved in child welfare or juvenile justice systems are likely to present with a range of intra- and interpersonal difficulties that are complicated by social and environmental factors contributing to health disparities, such as poverty, poor access to care, inadequate housing, health concerns, and unemployment. For example, parents in the child welfare system with disabilities are more likely to have inadequate housing as compared to parents without disabilities (Heyman et al., 2023). Parents in the child welfare system with substance use disorders present with a range of risk factors that can complicate the provision of treatment (Lloyd Sieger et al., 2023). There is also evidence of bidirectional impacts; for example, the involvement of female youth in the juvenile justice system has been found to have negative impacts on Black and Indigenous caregiver’s mental and physical health (Fix & Mendelson, 2022). Preparing service systems and staff to effectively support and intervene with parents who present with a complex and interacting array of challenges is a monumental task requiring substantial investments in training and ongoing supervision.

At a practical level, considering cost and time, service systems commonly offer group-based interventions despite evidence that this differs from a format of intervention desired by parents (Metzler et al., 2012). In addition, parents mandated for participation can differ in important ways from parents seeking services voluntarily; service systems thus need to have the capacity to offer group-based services separately for parents who are mandated to attend as compared to parents who are presenting voluntarily. While this is standard practice in mental health and other service settings, no research was located that empirically examined the processes or impacts of mixing mandated and voluntary clients in treatment settings.

Another challenge for service systems is supporting the quality of parenting intervention services provided, which impacts services for both voluntary clients and clients mandated for intervention. Indeed, service systems vary in the ability to implement evidence-based parenting interventions effectively (Barth et al., 2005; Edwards & Lutzker, 2008), which stand as the current “gold standard” of services in this area. This challenge is readily apparent in child welfare systems, where the adoption and implementation of evidence-based interventions have been noted to be slower than that of other service systems (Akin et al., 2015, 2016; Barth et al., 2005), likely due to the complex nature of such systems and multiple barriers to implementation (Garcia et al., 2019). However, despite these challenges, it is critical to elevate the quality of services for all families, mandated or not.

## Research on Impacts of Mandated Parenting Interventions

Given the challenges noted in the provision of mandated parenting programs, it is essential to examine the current empirical literature regarding the implementation and impacts of mandated parenting interventions across the service sectors most likely to provide these services—child welfare and juvenile justice.

### Child Welfare

Within the child welfare system, mandates for participating in parenting interventions are primarily utilized to address child maltreatment. Child maltreatment is an umbrella term for a global phenomenon inclusive of both child neglect and child psychological, physical, or sexual abuse. Uniform definitions of child maltreatment have been established and include deliberate words or overt actions that cause harm, potential harm, or threat of harm (i.e., physical abuse, sexual abuse, psychological abuse) and failure to provide for a child’s basic needs or to protect a child from harm or potential harm (i.e., physical, emotional, medical, educational neglect; Leeb et al., 2008). Global rates of child maltreatment types and how they can co-occur vary substantially based on the type of maltreatment and reporter (youth vs. adult) (Stoltenborgh et al., 2015). In the US, official national maltreatment rates (those reported to child welfare agencies and summarized at the federal level) typically identify neglect as the most common type of maltreatment. As an example, in 2022, a national rate of maltreatment of 7.7 victims per 1000 children was reported, with child neglect affecting the largest number of children at 74.3%; lower rates were found for physical abuse (17.0%), sexual abuse (10.6%), and psychological maltreatment (6.8%) (USDHHS, 2024). Parents are the most common perpetrators of child maltreatment (USDHHS, 2024), underscoring one reason for the focus on mandated parenting programs in this population. That said, when examining the research on parenting interventions in the child welfare system, we focused on studies for maltreatment generally and for neglect and physical abuse more specifically. We did not examine interventions for child sexual abuse, as this is a specialized area of intervention (Augarde & Rydon-Grange, 2022; Oliver & Washington, 2009); children in these cases are often removed from their homes or otherwise separated from these caretakers.

While there is a large body of research on parenting interventions used with families in the child welfare system, examining the impacts of mandated parenting programs is hampered by the lack of specification of parent status (mandated/involuntary vs. voluntary) in these studies. This is likely in part because child welfare interventions can be provided as voluntary, preventive services, as exemplified by the federal Title IV-E Prevention Program, known as the Family First Prevention Services Act (FFPSA) (https://www.acf.hhs.gov/cb/title-iv-e-prevention-program). FFPSA supports the use of federal funding for the implementation of evidence-based interventions to prevent child maltreatment and improve outcomes such as child behavior or parenting that are related to maltreatment prevention. That said, in cases where child maltreatment has been found, interventions can be mandated for parents as a condition of children remaining in the home or as a requirement before children are returned to the care of a parent(s). When the status of the participants in parenting studies is not explicit, it is difficult to locate studies and draw conclusions about the impact of interventions on a mandated population of parents.

Despite these drawbacks, some early studies were identified that explicitly examined outcomes for parents who were mandated to attend parenting interventions within the child welfare system. One early study of 42 families reviewed the impacts of a multi-faceted treatment program to address child maltreatment for parents who were participating voluntarily as compared to parents who were court-mandated to attend (Irueste-Montes & Montes, 1988). Both groups received the same parenting intervention, and data were analyzed separately; similar attendance patterns and changes in specific parenting skills (enhanced use of praise, reduction in criticisms) were noted in both groups of parents. Generalization of these results is limited as data were examined post-hoc. Similarly, a study examining the impacts of a social support skill training intervention for parents mandated to treatment in the child welfare system in Canada found no effects of the intervention as compared to a control condition; however, it should be noted that the study used a quasi-experimental design (Lovell & Richey, 1997).

More recent research on interventions for maltreatment has focused on specific types of maltreatment. A prime example is the effort to intervene in cases of child neglect, the largest category of maltreatment based on official reports in the US (USDHHS, 2024). That said, there are a limited number of interventions to address child neglect that have empirical support. One program, SafeCare, demonstrates the impact of factors related to child neglect. SafeCare is an 18-session behavioral parenting intervention for parents of children 0–5 delivered using a home-visitation model (Self-Brown et al., 2014). Studies document positive outcomes from the SafeCare program, including improvements in parenting behaviors and parenting stress (Whitaker et al., 2020), reductions in home safety hazards (Rostad et al., 2017), improvement in parenting skills in real-world implementation settings (Rogers-Brown et al., 2020), and statistical but not clinical impacts on caregiver well-being (anxiety/depression; Romano et al., 2020). However, impacts on neglect outcomes were not found (Whitaker et al., 2020). While these results are promising, specific information on whether these participants were mandated to participate in SafeCare is unclear, and implementation challenges, including lack of use of the model after training, have been noted (Whitaker et al., 2012).

While addressing child neglect is critical given the high prevalence rate of this type of maltreatment, focus on treating child physical abuse is also quite important, as is the identification of interventions that can be effective across multiple types of child maltreatment. Within child welfare populations, several evidence-based parenting intervention models were identified that appear to have positive impacts on parenting and youth functioning, including variations of Multisystemic Therapy (MST), a community-based, multi-component principle-driven intervention with a strong evidence base in treating youth with severe conduct problems (Henggeler, 2011, 2017), variations of the Triple P system of parenting interventions (Sanders & Kirby, 2014), and Parent–Child Interaction Therapy (PCIT; Eyberg et al., 2001).

A variant of MST called Multisystemic Therapy for Child Abuse and Neglect or MST-CAN was found in one randomized trial (comparing MST-CAN to enhanced outpatient treatment) to result in improvements in parent mental health symptoms, parenting behaviors, parenting supports, and placement outside of the home; instances of reabuse were lower but not statistically significant (Swenson et al., 2010). In a more recent study, another novel variant of MST called MST-Building Stronger Families (MST-BSF), was examined in an RCT with 98 parents in the child welfare system for abuse or neglect who also had substance misuse (Schaeffer et al., 2021). MST-BSF emphasizes intervention for adults in the family as well as child safety; as compared to a Comprehensive Community Treatment intervention, improvements in substance use and neglect were found, while reabuse was not significantly different between groups (Schaeffer et al., 2021). While both MST variants appear to have positive impacts on factors related to maltreatment, including parenting behaviors, statistically significant reductions in reabuse rates have not been achieved. In addition, as with other research studies of interventions with parents under the supervision of the child welfare system, parents appear to have been mandated to care, but this was not made explicit.

The Triple P system of interventions has significant empirical support with evidence of positive impacts on both parent and child outcomes (Sanders et al., 2014) and has been examined in child welfare populations (Lewis et al., 2016; Zhou et al., 2017). Pathways Triple P is an intensive variant that adds modules to the broad-based Triple P parenting intervention (i.e., Level 4 Triple P) to specifically address parent anger and maladaptive attributions for child behavior that can be related to child maltreatment (Wiggins et al., 2009). Lewis and colleagues examined the perception of Pathways Triple P for parents involved in the child welfare system in the USA, who reported that the content was valuable and relevant (Lewis et al., 2016); whether parents were mandated or not was not specified. In a clinical trial in Singapore with families referred (but not mandated) for intervention in the child welfare system, Zhou and colleagues found evidence of positive changes in parenting and child behaviors for broad-based parent training (Triple P Level 4), but likely due to attrition did not find impacts on parent anger or parent attributions targeted in Pathways Triple P (Zhou et al., 2017).

Another evidence-based parenting intervention, Parent–Child Interaction Therapy (PCIT), was created for parents of young children (ages 2–7) with significant behavioral problems (Eyberg & Matarazzo, 1980; Eyberg et al., 2001). PCIT has been since adapted to a broader range of presenting problems in children, including for parents where maltreatment has been identified. A review of 11 studies of PCIT with parents who have maltreated their children notes positive impacts on both parenting and child behaviors (Batzer et al., 2018). However, attrition was also noted in these studies and was a significant challenge (Batzer et al., 2018). PCIT has also been examined as an intervention for foster parents, who play a key role in supporting youth who have been removed from their homes due to child maltreatment. In a randomized study, 129 parent–child dyads were assigned to either a brief or extended version of PCIT or a waitlist comparison condition (Mersky et al., 2015); positive outcomes were found for self-reported parenting stress and observed parenting practices. However, whether these parents were required to attend the program was not reported.

In other research on interventions for foster parents, evidence of positive impacts of parenting interventions has been found. Still, similar to the research on the effects of parenting interventions for biological parents in the child welfare system, the status of the participants (mandated/involuntary or voluntary) is not reported. Benesh and Cui (2017) conducted a content review of 22 foster parent training programs and participants; however, this review did not include information on the outcomes assessed (Benesh & Cui, 2017). A meta-analysis of 16 studies on the impacts of foster parent training found small positive impacts on child behavior and moderate impacts on parenting knowledge and skills (Solomon et al., 2017). A large, universal, randomized trial of Multidimensional Treatment Foster Care (MTFC; Chamberlain, 2003) found positive changes in child behavior and parenting practices (Chamberlain et al., 2008). However, specific information on the status of parents in this trial (mandated vs. voluntary) was not provided.

Regardless of the treatment approach, a significant challenge noted in studies of parenting interventions for child maltreatment is attrition. High attrition rates dampen possible intervention impacts and challenge the ability to draw firm conclusions about the outcomes that can be achieved. Attrition was noted as a major challenge in reviewing PCIT studies on maltreatment (Batzer et al., 2018) and in the study of Triple P interventions by Zhou and colleagues (2017). A separate mixed-methods study with 31 parents mandated to parent education examined differences between parents who completed and those who did not complete the intervention; those who did not complete the intervention were reported to have higher levels of problematic parenting, parenting stress, and less social support (McWey et al., 2015). Attrition can thus be an important indicator of parenting challenges; parents most likely to stop participating may be those most in need.

In sum, there is empirical support for existing and novel interventions to have positive impacts on child maltreatment-related constructs, including parenting behaviors, yet impacts on rates of subsequent child maltreatment have yet to be found (e.g., Whitaker et al., 2020). In addition, attrition and poor engagement, even when interventions are delivered using home visitation models, are of concern (Guastaferro et al., 2020). Of note, the studies examined did not clearly and consistently report whether parents were mandated to participate in these interventions. To better understand treatment outcomes for parents who are mandated to attend parenting programs in the child welfare system, researchers are urged to consider more precise reporting of participant status (mandated vs. voluntary) in intervention studies, in line with calls to examine in more depth which programs and program elements work best for whom (Kemmis-Riggs et al., 2018). Within intervention studies, examining specific mechanisms of change based on parent status (mandated vs. voluntary) could also help move the field forward.

### Juvenile Justice

The intertwined realms of juvenile justice and legal reforms present a complex, dynamic interplay that significantly influences the efficacy of parenting interventions. At the heart of this nexus lies the recognition that the behaviors and outcomes of young individuals often reflect broader family and societal structures. Juvenile justice systems around the world are increasingly moving away from punitive approaches, recognizing the value of incorporating mental health perspectives to address the underlying issues that lead to delinquent behavior. The intersection of juvenile justice and parenting interventions is underpinned by a historical responsibility placed on parents for the criminal actions of their offspring, a concept dating back to the early twentieth century. This foundational belief has led to the evolution of various legal mechanisms aimed at correcting family dynamics believed to fuel juvenile delinquency (Burney & Gelsthorpe, 2008). In recent years, legislative frameworks, such as the “parenting order” in the UK, reflect a paradigm shift toward rehabilitation rather than punishment, compelling parents to engage in counseling or guidance sessions to rectify the underlying issues contributing to their child’s behavior (Burney & Gelsthorpe, 2008). Despite these legislative efforts, there remains a critical need for comprehensive research to evaluate the outcomes of mandated parenting interventions within the juvenile justice system. Existing literature predominantly focuses on the theoretical bases of court-ordered parenting skills (Schaffner, 1997) and qualitative assessments of niche groups, including adolescent fathers undergoing probation (Parra-Cardona et al., 2006, 2008).

The challenge of addressing juvenile delinquency and severe conduct issues among youth necessitates a multi-faceted, family-centered approach. Evidence-based interventions such as Multisystemic Therapy (MST) and Functional Family Therapy (FFT) have successfully improved family dynamics and reduced delinquency rates. MST, for instance, has shown that enhancing parenting skills is pivotal in moderating general delinquency outcomes (Henggeler, 2011; Henggeler & Schaeffer, 2016; van der Stouwe et al., 2014). Similarly, FFT has demonstrated effectiveness in mitigating conduct problems and substance use within juvenile justice and mental health settings, offering a promising alternative to traditional punitive measures or less structured treatments (Hartnett et al., 2017). Multisystemic Therapy (MST) and Functional Family Therapy (FFT) stand out for their reliance on bolstering parenting skills as a cornerstone for intervention. MST offers a holistic, community-based strategy that addresses the myriad factors contributing to antisocial behavior in youth, demonstrating a significant reduction in long-term criminal activities among participants (Henggeler et al., 2009). Fundamental to MST’s success is empowering parents with effective boundary-setting, fostering family connections, and promoting problem-solving skills. FFT echoes this by emphasizing the transformation of family dynamics and communication to curb youth delinquency and substance misuse. Research substantiates FFT’s efficacy in mitigating behavioral issues, underscoring the importance of nurturing positive family interactions as a preventive measure against juvenile delinquency (Sexton & Turner, 2010). However, a notable gap persists in the research concerning the outcomes of these interventions under mandated circumstances, particularly in evaluating the modifications in parenting techniques and the broader implications for family and youth development (Henggeler et al., 2009; Sexton & Turner, 2010). Studies should aim to quantify changes in parenting practices post-intervention and assess these changes’ correlation with improved child outcomes. Additionally, exploring the long-term sustainability of such interventions and their ability to foster positive family dynamics warrants attention. Addressing these research gaps could provide invaluable insights into optimizing juvenile justice strategies to support at-risk families more effectively.

## Advancing Research on Mandated Parenting Programs

Advancing research on mandated parenting programs across service systems, including child welfare and juvenile justice, requires that studies more clearly document the nature of the intervention, i.e., whether it is mandated or voluntary. Existing evidence-based parenting interventions have demonstrated the ability to change parenting behaviors and youth outcomes (e.g., SafeCare, PCIT, Triple P, MST) and likely included parents mandated to interventions. However, this information needs to be made explicit in published studies to improve our understanding of outcomes for parents specifically mandated to intervention. Furthermore, research that focuses on mechanisms of change in the context of mandated interventions is required, including comparative studies that examine whether these mechanisms operate in similar or different ways in voluntary as compared to mandated samples of participants. Moving the field forward will require an increase in the number of well-conducted studies focusing specifically on parent and child outcomes within mandated interventions in real-world service settings. At the same time, additional, high-quality research is needed to examine the pathways and impacts of mandated parenting interventions across service systems more clearly, and alternative approaches to supporting parents in a less adversarial way must also be considered.

## Reducing the Need for Mandated Parenting Programs

To address the challenges of mandated services, the provision of universal parenting support for all parents of children or youth within a specified target population offers a unique opportunity to strengthen parenting that, by design, includes families at risk for child/adolescent behavior problems and/or parenting challenges that can lead to eventual involvement in child welfare, juvenile justice, mental health, and other service systems. Conceptualizing parenting from a public health perspective offers both a conceptual framework and a pathway to providing tiered parenting support, as noted by Salem and colleagues in the context of divorce within family court systems (Salem et al., 2013). The provision of universal parenting support using a tiered public health model is a powerful method that can potentially reduce the number of parents mandated to participate in parenting interventions; mandated programs could then be reserved for a smaller number of parents in specific situations requiring a more intensive approach.

Viewing parenting through a public health lens is most clearly illustrated in the Triple P model of intervention that explicitly utilizes a public health framework and consists of a multi-level system of parenting supports (Sanders, 2008, 2012; Sanders & Kirby, 2014; Sanders et al., 2008, 2014). Aiming to reach the broadest number of parents possible with evidence-based parenting supports, Triple P interventions are built as a tiered system designed to provide the minimally sufficient level of support necessary for positive child and parent outcomes to be achieved (Sanders & Kirby, 2014). Consistent with public health models, Triple P interventions are grounded in population-wide communication strategies to promote positive parenting, with increasing intensity available based on needs and parent desires (Sanders, 2012). Indeed, the universal offering of parenting information through non-traditional communication vehicles, such as televised series, has been found to positively impact parenting and child outcomes (e.g., Calam et al., 2008; Sanders et al., 2008).

Relevant to our focus on alternatives to mandated parenting approaches, evidence of positive impacts on constructs related to child maltreatment has been found in a place-based population-level randomized trial of Triple P interventions targeting all parents of children below age 8 residing in specific counties in one state in the Southern U.S. (Prinz et al., 2009). A total of 18 counties within this state were matched in pairs on key characteristics (population size, poverty rates, and rates of child maltreatment); one county within each pair was then randomly assigned to receive the tiered Triple P system of interventions. The remaining counties provided services as usual. Positive impacts were found on important indicators of child maltreatment at a population level, including rates of out-of-home placements in the foster care system, hospitalizations for maltreatment-related injuries, and rates of founded cases of child maltreatment (Prinz et al., 2009, 2016).

Beyond child maltreatment-related outcomes, population-wide parenting supports targeting parents of young children ages 2–5 have been used successfully in Scotland to support positive child development. In a national implementation of two evidence-based parenting interventions, Group Triple P and Incredible Years Preschool Basic, large pre-post reductions in child behavior challenges were found (Saunders et al., 2020). Thus, evidence is growing that providing parenting support to a large population of parents can have important impacts on outcomes related to child development and, ultimately, on child maltreatment. The provision of widespread support focusing on parents of young children, as in the studies by Prinz and colleagues (2009) and Saunders and colleagues (2020), is particularly important from a prevention standpoint.

While population-level interventions show promise to achieve changes in important maltreatment and behavior outcomes, and putative (but not empirically examined) mechanisms of change have been identified (Sanders & Mazzucchelli, 2022), implementation of these types of interventions is a complex endeavor (Shapiro et al., 2010) and requires substantial resources. A feasible alternative to reach large groups of parents includes universally available, brief large-group interventions, including parenting seminars; studies have demonstrated that these types of interventions can result in positive changes in child behavior, parenting practices, and parent stress (Foskolos et al., 2023; Sumargi et al., 2015). Offering brief, single-session interventions for large groups of parents can destigmatize help-seeking and provide minimally sufficient support to create changes in important outcomes related to the reasons for using mandated parenting interventions. Indeed, a tiered approach offering universal and targeted parenting support to address income-related disparities in school readiness has effectively increased attendance (Canfield et al., 2023). In addition, online parenting programs that are evidence-based and made widely available offer another avenue to increase parents’ access and to possibly reduce the need for mandated interventions in the future. Indeed, research on online parenting interventions proliferates and positive impacts on parents/parenting as well as child behavior have been found (Baker et al., 2017; Baumel & Faber, 2018; Day & Sanders, 2017, 2018; Lappalainen et al., 2023).

Additional avenues to reach large numbers of parents with timely and effective, evidence-based parenting interventions include incorporating parenting support into a range of existing service settings where parents are naturally found, such as early care and education, health care, and schools. Using an existing workforce, as demonstrated in the Triple P Population Trial (Prinz et al., 2009, 2016), can eliminate barriers to providing parenting support in an era of substantial shortages in mental health professionals (Hunt et al., 2019). Advocating for models that co-locate parenting programs with mental health and substance abuse treatment services is another strategy that can minimize access barriers and foster more efficient service provision and better outcomes for families by offering a more coordinated approach to care (Huebner et al., 2021). Implementing universal screening within pediatric and primary care settings can help to identify parents requiring additional support; this proactive approach facilitates timely interventions, addressing potential issues before they escalate (Council on Children with Disabilities & Medical Home Implementation Project Advisory Committee, 2014). While these approaches to incorporating parenting support into existing service systems have tremendous potential, additional reforms are warranted to further enhance support for parents and children. Funding for legal initiatives to boost mental health and substance abuse services is vital, as are policy-level changes to enhance parent access to evidence-based supports (Doyle et al., 2023). Such reforms would ensure all families have access to care, alleviate financial burdens, and promote overall well-being (Council on Children with Disabilities & Medical Home Implementation Project Advisory Committee, 2014).

While there are existing methods to broadly disseminate parenting support, which can impact parent and child behavior, one major drawback is that we do not know how such universal strategies for parenting support impact the number of parents mandated to participate in parenting programs. This is an empirical question that remains unanswered. To help address this question, we must first consider that no single source exists for establishing the number of parents mandated to parenting programs on an annual basis. Mandates cross service sectors, which complicates our ability to obtain a clear count; even within service sectors, it is not currently possible to obtain exact counts of numbers of parents mandated to attend these programs. Thus, well-developed data systems must be established to track the number of parents mandated to parenting programs. In addition, studies of universal parenting supports using longitudinal designs are needed to detect the downstream effects on the number of families who become involved in juvenile justice or child welfare systems—systems most likely to mandate parenting programs. Moving the field forward will require strong cross-system collaboration and substantial investments by funding organizations at the state and/or federal level to support the most needed studies.

## Future Directions and Promising Practices

Recently, mandates for parents to participate in parenting programs have expanded into early childhood/school readiness programs. Various early childhood settings exist for young children, such as public school programs (i.e., publicly funded preschool programs and/or special education preschool programs), Head Start initiatives, private preschools, early intervention, and/or childcare programs. These settings offer a promising opportunity to provide population-based parenting support during early childhood as primary prevention. Parent education initiatives available through these venues can vary widely, encompassing home visitation programs, parent workshops, and parent education classes.

For instance, Head Start requires the integration of parent and family engagement strategies into all systems and program services to support family well-being and promote children’s learning and development. Programs are encouraged to include a “research-based parenting curriculum” to enhance child learning and development (refer to: https://eclkc.ohs.acf.hhs.gov/policy/45-cfr-chap-xiii/1302-51-parent-activities-promote-child-learning-development). This encompasses diverse opportunities for parental involvement, tailored to augment parents’ existing skills. However, participation is voluntary, and parents can select their preferred involvement method, such as engaging with teachers or volunteering in the classroom (Head Start Performance Standards, Subpart E 1302, U.S. Department of Health and Human Services, 2016).

Organizations dedicated to school readiness for children from birth to age 5 and their families exist in most states. Examples include Smart Start in North Carolina and First Five in California, offering various programs for child and family support for children at risk of academic difficulties due to factors like low income or parental education level. Programs typically include a range of services, including home visitation (e.g., Parents as Teachers), kindergarten transition, early education and care access, and childcare cost subsidies (Forry et al., 2013; Lahti et al., 2019). Importantly, due to financial constraints, Head Start and school readiness supports are typically made available only to families with limited financial resources or other risk factors for poor academic outcomes; thus, parenting support embedded in these systems will reach only a portion of the population.

Similarly, the Individuals with Disabilities Education Act (IDEA) Part C outlines services for infants and toddlers who have or are at risk for developmental delays. These services require a family-centered approach and the creation of an Individual Family Service Plan (IFSP). The IFSP identifies the family’s resources, concerns, goals, and the necessary support for the child’s development (U.S. Department of Education, 2004). While these interventions are mandatory, their exact nature is not specified; ideally, they should be based on evidence-based models and peer-reviewed research. There are few studies, but reviews suggest that the outcomes are generally positive, with specific parent education interventions showing promise (Bates, 2005).

Within early childhood populations, research supports the effectiveness of parent education and training programs for parents of preschoolers, especially those with ADHD and behavioral challenges (e.g., PCIT, Eyberg et al., 2001). Programs such as the *New Forest Parenting Programme* and *Helping the Noncompliant Child* have demonstrated positive outcomes in reducing behavior problems, improving parent mental health and skills, and lessening harmful parenting practices (Abikoff et al., 2015; Barlow & Coren, 2018; Lange et al., 2018; Rimestad et al., 2019). Moreover, the success of these programs extends to community preschool settings, exemplified by the *Parent Plus Early Years Programme*. The evidence base also includes self-guided interventions like *Promoting Resilience for Now and Forever* and those aimed at fathers, such as the COACHES program (Caserta et al., 2018; Gerber et al., 2016; Thomson & Carlson, 2017). However, while Barlow and Coren’s review of parenting programs reveals reduced behavior problems, improved parental mental health, and decreased use of harmful parenting practices for children aged 3–12, the evidence is limited for children aged 0–3 (Barlow & Coren, 2018). While findings of impact in these studies in early childhood populations predominantly come from parent reports, with variable results from other sources, Axford et al. (2019) further corroborated the positive impact of parental involvement in early childhood programs. Concerning younger children, studies on Early Head Start highlight the value of parenting classes in fostering better cognitive and linguistic stimulation, improving parent–child interactions, and advancing cognitive development (Chang et al., 2009). Notably, these classes were optional, and whether compulsory attendance would yield similar benefits remains to be determined.

In a systematic review of 26 studies, Butler et al. (2020) examined parent perceptions and experiences with parenting programs, illuminating the challenges parents face before entering such programs—e.g., dealing with children’s behavioral issues, feeling disconnected from their children, having distressing interactions, and experiencing isolation. These feelings were particularly intense among parents who were mandated to attend as part of child welfare interventions. Despite some initial skepticism or reluctance toward the programs, many parents described a significant shift toward willingness and active engagement. They reported learning and reinforcing parenting skills, such as strategies for emotional regulation, that helped reduce negative interactions like shouting or physical punishment. These skills fostered a more positive and empowering parenting experience, improving parent–child relationships, enhanced parenting confidence, and a feeling of regaining control. The improvement was not just in parent well-being; parents also observed better behavior in their children and an overall enhancement in family life. The shift from pessimism to willingness and the acquisition of new parenting skills highlight the transformative potential of these parenting programs (Butler et al., 2020).

While enhancing the current outreach of parenting programs is crucial, it is equally important to ensure that these initiatives are accessible and relevant to those who need them most. To this end, primary care settings emerge as another promising avenue for embedding parenting support, reaching a broader demographic, and facilitating early intervention and support for child development and well-being. Primary care settings can serve as a venue for screening and possible intervention for maternal depression, an important risk factor for poor child mental health outcomes (Engelhard et al., 2022). Integrating behavioral health care for young children and their parents in pediatric primary care settings can reduce systematic inequities in access to care and parenting support (Margolis et al., 2022). When behavioral health care is collocated with pediatric primary care, evidence supports parent uptake (as measured by first-session attendance) and clinical improvement (Valleley et al., 2020). These positive and promising findings suggest that when behavioral health care and pediatric care are combined, access to parental support can increase and have positive benefits. 

In addition to pediatric and primary care settings, embedding parenting supports within community mental health and substance use treatment settings offers another avenue to reach large numbers of parents who may benefit from parenting support. The integration of these programs with mental health and substance abuse services has emerged as a critical, comprehensive approach to addressing the intricate relationship between parenting challenges, mental health issues, and substance misuse. This holistic strategy, which includes adaptable services like the Family-Centered Treatment (FCT) framework, emphasizes in-home care that spans mental health support, substance abuse treatment, and parenting education, proving effective across mandated and voluntary contexts. The personalized delivery of the FCT model caters to individual family needs, enhancing engagement and outcomes by providing a non-stigmatizing environment that encourages participation (Green et al., 2016).

The multidisciplinary approach, exemplified by models such as the Coordinated Family Care (CFC), unites mental health professionals, substance abuse specialists, and parenting experts to create a seamless continuum of care. This unified strategy is particularly advantageous for mandated programs by simplifying service navigation and ensuring continuity of care, thus reducing barriers to access and fostering environments conducive to stability and nurturing (Duan-Porter et al., 2020). The incorporation of such interventions within the larger framework of legal and service-based reforms, as seen in the European Union with CAMHEE (Child and Adolescent Mental Health European Education; Braddick et al., 2009) and in the U.S. with the Family First Prevention Services Act, aligns with the global movement toward preventative care and integrated support services to enhance family cohesion and stability (Braddick et al., 2009; Lindell et al., 2020).

Parent education programs in these settings influence family dynamics, facilitate positive outcomes such as parenting behaviors and child adjustment, and help foster a nurturing and supportive family environment, particularly when paired with increased parental self-efficacy. However, the effective implementation of these programs must navigate the challenges of high dropout rates and the unique complexities of families facing socioeconomic constraints, substance abuse, and mental health issues. Targeted support and specialized training for caseworkers are essential in addressing these challenges, ensuring that interventions are tailored, flexible, and accessible to all families, particularly those engaged in mandated programs (Akin & Gomi, 2017; Gewirtz et al., 2009; Staudt & Cherry, 2009).

These models showcase the benefits of integrated, comprehensive strategies for supporting families, ensuring that interventions align with the complex needs of those enrolled in mandated and voluntary programs. Such integrated services are critical for achieving improved outcomes, demonstrating the essential role of coordinated care in developing resilient and cohesive family units. Implementing these models represents a progressive step in enhancing the efficacy of parenting programs as part of a broader mental health and substance abuse prevention framework, ensuring families receive the support they need to thrive. These models, illustrating the effectiveness of integrated, comprehensive strategies, ensure that interventions are relevant to the complex needs of families involved in mandated and voluntary programs. Integrated services are crucial in achieving improved outcomes and developing resilient and cohesive family units. Such implementation is a progressive step in the efficacy of parenting programs as part of a broader mental health and substance abuse prevention framework, guaranteeing that families receive the support they need to flourish.

In community mental health settings, the unique and diverse challenges that families face require intervention strategies that are specifically designed to be flexible and personalized. These strategies must be adaptable enough to cater to the stringent requirements of mandated programs while remaining suitable for families seeking voluntary services. Such customized interventions, which include provisions like flexible scheduling and teletherapy, are crucial in enhancing the accessibility and effectiveness of support for all families, making it possible to align services with individual family dynamics and increase the potential for successful outcomes. Furthermore, culturally sensitive materials can be integrated into these interventions to build trust and ensure meaningful engagement. They are critical for overcoming resistance, particularly within mandated programs, by reflecting the families’ cultural experiences in the provided services (Liu et al., 2005). The implementation process, which requires a deep understanding of each family’s situation, may involve detailed assessments and regular feedback mechanisms to adjust the support provided. Mental health professionals also need ongoing training to ensure the delivery of culturally competent and responsive care (Osman et al., 2021; Liu et al., 2005). Additionally, service providers also need to be prepared to assist parents in overcoming barriers to service engagement (especially pertinent for parents in mandated parenting programs) where external factors such as socioeconomic constraints can hinder participation. Addressing these challenges with targeted support—such as transportation assistance, childcare, motivational interviewing techniques, and the seamless integration of mental health and substance abuse treatments into parenting programs—is crucial for ensuring that all families, regardless of their circumstances, can fully engage with and benefit from these services (Mytton et al., 2014; Miller & Rollnick, 2013).

This comprehensive approach is supported by legal and service-based reforms such as the Family First Prevention Services Act in the USA and the Troubled Families Programme in the UK, which have proven essential in enhancing the well-being of families dealing with mental health and substance abuse challenges. These reforms have underlined the efficacy of combining legislative support with flexible, tailored interventions to create an effective, responsive family support system (Ministry of Housing, Communities, & Local Government, 2021; Pritzker & Smith, 2021). As the juvenile justice, community mental health, primary care, and education sectors intersect, it becomes evident that integrating parenting programs with mental health and substance abuse services is vital for forming a strong support network. This network is designed to confront the complex issues that today’s families encounter more effectively. Continuous research into their long-term effects is essential to maximize the impact of these interventions, particularly for mandated parenting programs. Such research will inform the refinement of intervention implementation and contribute to a better understanding the developmental outcomes for families and youth.

## Conclusion

Mandates for parents to attend parenting interventions are common for parents who are divorcing, as well as for parents in child welfare and juvenile justice settings, affecting many parents each year, but data on the exact number of parents impacted is lacking. Mandates to participate in parenting programs present substantial challenges for parents, providers, and service systems. For families mandated to parenting programs, the number of high-quality studies that carefully document the status of parents as voluntary or mandated is far less than would be helpful to determine impacts specifically for parents who are mandated to intervention. Universal provision of parenting supports has demonstrated positive impacts on child maltreatment, parenting, and child outcomes, yet the mechanisms of action for these large-scale studies require empirical support. We also do not know if provision of universal supports can reduce the number of parents who are mandated to parenting programs. To address these questions, population-level longitudinal research designs are needed, buttressed by sophisticated cross-system data collection that allows the impacts of parenting supports to be traced over time and across service systems. While impacts on mandated parenting programs have not yet been documented, providing population-wide parenting support through a range of community service settings and innovative use of self-help and communication vehicles can prevent and treat child behavior and common parenting challenges. These powerful alternative approaches demonstrate promise to reduce the need for mandated approaches to parenting interventions, but this still needs to be realized.

## References

[CR1] Abikoff HB, Thompson M, Laver-Bradbury C, Long N, Forehand RL, Miller Brotman L, Klein RG, Reiss P, Huo L, Sonuga-Barke E (2015). Parent training for preschool ADHD: A randomized controlled trial of specialized and generic programs. Journal of Child Psychology and Psychiatry.

[CR2] Akin BA, Brook J, Byers KD, Lloyd MH (2015). Worker perspectives from the front line: Implementation of evidence-based interventions in child welfare settings. Journal of Child and Family Studies.

[CR3] Akin BA, Brook J, Lloyd MH, Bhattarai J, Johnson-Motoyama M, Moses M (2016). A study in contrasts: Supports and barriers to successful implementation of two evidence-based parenting interventions in child welfare. Child Abuse and Neglect.

[CR4] Akin BA, Gomi S (2017). Noncompletion of evidence-based parent training: An empirical examination among families of children in foster care. Journal of Social Service Research.

[CR5] Augarde S, Rydon-Grange M (2022). Female perpetrators of child sexual abuse: A review of the clinical and empirical literature—A 20-year update. Aggression and Violent Behavior.

[CR6] Axford, N., Berry, V., Lloyd, J., Moore, D., Rogers, M., Hurst, A., Blockley, K., Durkin, H., & Minton, J. (2019). *How can schools support parents’ engagement in their children’s learning? Evidence from research and practice*. Education Endowment Foundation. Retrieved from https://educationendowmentfoundation.org.uk/evidence-summaries/evidencereviews/parental-engagement/

[CR7] Baker S, Sanders MR, Turner KMT, Morawska A (2017). A randomized controlled trial evaluating a low-intensity interactive online parenting intervention, Triple P Online Brief, with parents of children with early onset conduct problems. Behaviour Research and Therapy.

[CR8] Barlow J, Coren E (2018). The effectiveness of parenting programs: A review of Campbell reviews. Research on Social Work Practice.

[CR10] Barth RP, Landsverk J, Chamberlain P, Reid JB, Rolls JA, Hurlburt MS, Farmer EMZ, James S, McCabe KM, Kohl PL (2005). Parent-training programs in child welfare services: Planning for a more evidence-based approach to serving biological parents. Research on Social Work Practice.

[CR11] Bates SL (2005). Evidence-based family-school interventions with preschool children. School Psychology Quarterly.

[CR12] Batzer S, Berg T, Godinet MT, Stotzer RL (2018). Efficacy or chaos? Parent-child interaction therapy in maltreating populations: A review of research. Trauma, Violence, and Abuse.

[CR13] Baumel A, Faber K (2018). Evaluating Triple P Online: A digital parent training program for child behavior problems. Cognitive and Behavioral Practice.

[CR14] Becker KD, Chorpita BF (2023). Future directions in youth and family treatment engagement: Finishing the bridge between science and service. Journal of Clinical Child and Adolescent Psychology.

[CR15] Becker KD, Lee BR, Daleiden EL, Lindsey M, Brandt NE, Chorpita BF (2015). The common elements of engagement in children’s mental health services: Which elements for which outcomes?. Journal of Clinical Child and Adolescent Psychology.

[CR16] Benesh AS, Cui M (2017). Foster parent training programmes for foster youth: A content review. Child and Family Social Work.

[CR17] Bowers JR, Mitchell ET, Hardesty JL, Hughes R (2011). A review of online divorce education programs. Family Court Review.

[CR18] Bowers JR, Ogolsky BG, Hughes R, Kanter JB (2014). Coparenting through divorce or separation: A review of an online program. Journal of Divorce and Remarriage.

[CR19] Braddick F, Carral V, Jenkins R, Jané-Llopis E (2009). Child and Adolescent Mental Health in Europe (CAMHEE): Infrastructures, policy and programmes.

[CR20] Burke JD, Mulvey EP, Schubert CA, Garbin SR (2014). The challenge and opportunity of parental involvement in juvenile justice services. Children and Youth Services Review.

[CR21] Burney E, Gelsthorpe L (2008). Do we need a “naughty step”? Rethinking the parenting order after ten years. Howard Journal of Criminal Justice.

[CR22] Butler J, Gregg L, Calam R, Wittkowski A (2020). Parents’ perceptions and experiences of parenting programmes: A systematic review and metasynthesis of the qualitative literature. Clinical Child and Family Psychology Review.

[CR23] Calam R, Sanders MR, Miller C, Sadhnani V, Carmont S-A (2008). Can technology and the media help reduce dysfunctional parenting and increase engagement with preventative parenting interventions?. Child Maltreatment.

[CR24] Canfield CF, Miller EB, Zhang Y, Shaw D, Morris P, Galan C, Mendelsohn AL (2023). Tiered universal and targeted early childhood interventions: Enhancing attendance across families with varying needs. Early Childhood Research Quarterly.

[CR25] Caserta A, Fabiano GA, Hulme K, Pyle K, Isaacs L, Jerome S (2018). A waitlist-controlled trial of behavioral parent training for fathers of preschool children. Evidence-Based Practice in Child and Adolescent Mental Health.

[CR26] Chamberlain P (2003). The Oregon multidimensional treatment foster care model: Features, outcomes, and progress in dissemination. Cognitive and Behavioral Practice.

[CR27] Chamberlain P, Price J, Leve LD, Laurent H, Landsverk JA, Reid JB (2008). Prevention of behavior problems for children in foster care: Outcomes and mediation effects. Prevention Science.

[CR28] Chang M, Park B, Kim S (2009). Parenting classes, parenting behavior, and child cognitive development in early head start: A longitudinal model. School Community Journal.

[CR29] Turchi RM, Antonelli RC, Norwood KW, Adams RC, Brei TJ, Burke RT, Davis BE, Friedman SL, Houtrow AJ, Kuo DZ, Levy SE, Wiley SE, Kalichman MA, Murphy NA, Carl Cooley W, Jeung J, Johnson B, Klitzner TS, Lail JL, Lindeke LL, Mullins A, Partridge L, Council on Children with Disabilities and Medical Home Implementation Project Advisory Committee (2014). Patient- and family-centered care coordination: A framework for integrating care for children and youth across multiple systems. Pediatrics.

[CR30] David OA, Iuga IA, Miron IS (2024). Parenting: There is an app for that A systematic review of parenting interventions apps. Children and Youth Services Review.

[CR31] Day JJ, Sanders MR (2017). Mediators of parenting change within a web-based parenting program: Evidence from a randomized controlled trial of Triple P Online. Couple and Family Psychology: Research and Practice.

[CR32] Day JJ, Sanders MR (2018). Do parents benefit from help when completing a self-guided parenting program online? A randomized controlled trial comparing Triple P Online with and without telephone support. Behavior Therapy.

[CR33] Dinkmeyer D (1999). Working with court-ordered and mandated parents. The Journal of Individual Psychology.

[CR35] Doyle FL, Morawska A, Higgins DJ, Havighurst SS, Mazzucchelli TG, Toumbourou JW, Middeldorp CM, Chainey C, Cobham VE, Harnett P, Sanders MR (2023). Policies are needed to increase the reach and impact of evidence-based parenting supports: A call for a population-based approach to supporting parents, children, and families. Child Psychiatry and Human Development.

[CR36] Duan-Porter, W., Ullman, K., Majeski, B., Miake-Lye, I., Diem, S., & Wilt, T. J. (2020). *Care coordination models and tools: A systematic review and key informant interviews* [Internet]. Department of Veterans Affairs (US). Retrieved from https://www.ncbi.nlm.nih.gov/books/NBK566155/33400451

[CR37] Edwards A, Lutzker JR (2008). Iterations of the SafeCare Model, an evidence-based child maltreatment prevention program. Behavior Modification.

[CR38] Engelhard C, Hishinuma E, Rehuher D (2022). The impact of maternal depression on child mental health treatment and models for integrating care: A systematic review. Archives of Women’s Mental Health.

[CR39] Eyberg SM, Funderburk BW, Hembree-Kigin TL, McNeil CB, Querido JG, Hood KK (2001). Parent-child interaction therapy with behavior problem children: One and two-year maintenance of treatment effects in the family. Child and Family Behavior Therapy.

[CR40] Eyberg SM, Matarazzo RG (1980). Training parents as therapists: A comparison between individual parent-child interaction training and parent group didactic training. Journal of Clinical Psychology.

[CR41] *Fast Facts: Preventing child abuse & neglect|Violence Prevention|Injury Center*. (2023). CDC. Retrieved from https://www.cdc.gov/violenceprevention/childabuseandneglect/fastfact.html

[CR42] Fix RL, Mendelson T (2022). Stress, worry, and health problems experienced by Black and Indigenous caregivers of girls with juvenile legal system involvement. Children and Youth Services Review.

[CR43] Forry ND, Daneri P, Howarth G (2013). Child care subsidy literature review. OPRE Brief 2013-60.

[CR44] Foskolos K, Gardner F, Montgomery P (2023). Brief parenting seminars for preventing child behavioral and emotional difficulties: A pilot randomized controlled trial. Journal of Child and Family Studies.

[CR45] Garcia AR, DeNard C, Morones SM, Eldeeb N (2019). Mitigating barriers to implementing evidence-based interventions in child welfare: Lessons learned from scholars and agency directors. Children and Youth Services Review.

[CR46] Gerber S-J, Sharry J, Streek A (2016). Parent training: Effectiveness of the Parents Plus Early Years programme in community preschool settings. European Early Childhood Education Research Journal.

[CR47] Gewirtz AH, DeGarmo DS, Plowman EJ, August G, Realmuto G (2009). Parenting, parental mental health, and child functioning in families residing in supportive housing. American Journal of Orthopsychiatry.

[CR48] Goodman SH, Rouse MH, Connell AM, Broth MR, Hall CM, Heyward D (2011). Maternal depression and child psychopathology: A meta-analytic review. Clinical Child and Family Psychology Review.

[CR50] Green JG, Xuan Z, Kwong L, Anderson JA, Leaf PJ (2016). School referral of children with serious emotional disturbance to systems of care: Six-month clinical and educational outcomes. Journal of Child and Family Studies.

[CR51] Guastaferro K, Self-Brown S, Shanley JR, Whitaker DJ, Lutzker JR (2020). Engagement in home visiting: An overview of the problem and how a coalition of researchers worked to address this cross-model concern. Journal of Child and Family Studies.

[CR52] Hartnett D, Carr A, Hamilton E, O’Reilly G (2017). The effectiveness of functional family therapy for adolescent behavioral and substance misuse problems: A meta-analysis. Family Process.

[CR53] Havighurst S, Kehoe C (2017). The role of parental emotion regulation in parent emotion socialization: Implications for intervention. Parental stress and early child development: Adaptive and maladaptive outcomes.

[CR54] Henggeler SW (2011). Efficacy studies to large-scale transport: The development and validation of multisystemic therapy programs. Annual Review of Clinical Psychology.

[CR55] Henggeler SW (2017). Multisystemic therapy. The encyclopedia of juvenile delinquency and justice.

[CR56] Henggeler SW, Schaeffer CM (2016). Multisystemic Therapy^®^: Clinical overview, outcomes, and implementation research. Family Process.

[CR57] Henggeler SW, Schoenwald SK, Borduin CM, Rowland MD, Cunningham PB (2009). Multisystemic therapy for antisocial behavior in children and adolescents.

[CR58] Heyman M, Li F, Swinford L, Mitra M (2023). Housing circumstances of disabled parents within the child welfare system. Children and Youth Services Review.

[CR59] Hock R, Yingling ME, Kinsman A (2015). A parent-informed framework of treatment engagement in group-based interventions. Journal of Child and Family Studies.

[CR60] Huebner RA, Willauer T, Hall MT, Poole V, Posze L, Hibbeler PG (2021). Comparative outcomes for Black children served by the Sobriety Treatment and Recovery Teams program for families with parental substance abuse and child maltreatment. Journal of Substance Abuse Treatment.

[CR61] Hunt SM, Denby RW, Hertlein KM, Lefforge N, Paul MG (2019). A university-based transdisciplinary approach to mental health workforce shortages. Community Mental Health Journal.

[CR62] Irueste-Montes AM, Montes F (1988). Court-ordered vs voluntary treatment of abusive and neglectful parents. Child Abuse and Neglect.

[CR63] Kemmis-Riggs J, Dickes A, McAloon J (2018). Program components of psychosocial interventions in foster and kinship care: A systematic review. Clinical Child and Family Psychology Review.

[CR64] Kempe CH, Silverman FN, Steele BF, Droegemueller W, Silver HK (1962). The battered-child syndrome. JAMA.

[CR65] Kramer L, Washo CA (1993). Evaluation of a court-mandated prevention program for divorcing parents: The Children First program. Family Relations: An Interdisciplinary Journal of Applied Family Studies.

[CR66] Lahti M, Evans CBR, Goodman G, Schmidt MC, LeCroy CW (2019). Parents as Teachers (PAT) home-visiting intervention: A path to improved academic outcomes, school behavior, and parenting skills. Children and Youth Services Review.

[CR67] Lange AM, Daley D, Frydenberg M, Houmann T, Kristensen LJ, Rask C, Sonuga-Bark E, Søndergaard-Baden S, Udupi A, Thomsen PH (2018). Parent training for preschool ADHD in routine, specialist care: A randomized controlled trial. Journal of the American Academy of Child and Adolescent Psychiatry.

[CR68] Lappalainen P, Gallego A, Keinonen K, Lappalainen A-L, Tolvanen A, Lappalainen R (2023). Online and self-help acceptance and commitment therapy for parents of children with chronic conditions and developmental disabilities: What happens after the intervention?. Child and Family Behavior Therapy.

[CR69] Leeb, R. T., Paulozzi, L., Melanson, C., Simon, T., & Arias, I. (2008). *Child maltreatment surveillance: Uniform definitions for public health and recommended data elements, Version 1.0*. Centers for Disease Control and Prevention, National Center for Injury Prevention and Control.

[CR70] Lewis EM, Feely M, Seay KD, Fedoravicius N, Kohl PL (2016). Child welfare involved parents and pathways Triple P: Perceptions of program acceptability and appropriateness. Journal of Child and Family Studies.

[CR71] Lindell KU, Sorenson CK, Mangold SV (2020). The Family First Prevention Services Act: A new era of child welfare reform. Public Health Reports.

[CR72] Liu M, Chen X, Rubin KH, Zheng S, Cui L, Li D, Chen H, Wang L (2005). Autonomy- vs. connectedness-oriented parenting behaviours in Chinese and Canadian mothers. International Journal of Behavioral Development.

[CR73] Lloyd Sieger M, Becker J, Philips J, Lee JW, Moore TE (2023). Latent classes among substance-involved families in child welfare: Associations with treatment completion and reunification. Children and Youth Services Review.

[CR74] Loo J (2023). Divorce rate in the U.S.: Geographic variation, 2022. Family Profiles, FP-23-24.

[CR75] Lovell ML, Richey CA (1997). The impact of social support skill training on daily interactions among parents at risk for child maltreatment. Children and Youth Services Review.

[CR76] Manchak SM, Skeem JL, Rook KS (2014). Care, control, or both? Characterizing major dimensions of the mandated treatment relationship. Law and Human Behavior.

[CR77] Margolin G, Patterson GR (1975). Differential consequences provided by mothers and fathers for their sons and daughters. Developmental Psychology.

[CR78] Margolis KL, Buchholz M, Charlot-Swilley D, Serrano V, Herbst R, Meiselman E, Talmi A (2022). Early childhood integrated behavioral health: A promoter of equity in pediatric care. Clinical Practice in Pediatric Psychology.

[CR79] McKenry PC, Clark KA, Stone G (1999). Evaluation of a parent education program for divorcing parents. Family Relations: An Interdisciplinary Journal of Applied Family Studies.

[CR80] McWey LM, Holtrop K, Wojciak AS, Claridge AM (2015). Retention in a parenting intervention among parents involved with the child welfare system. Journal of Child and Family Studies.

[CR81] Mersky JP, Topitzes J, Janczewski CE, McNeil CB (2015). Enhancing foster parent training with parent-child interaction therapy: Evidence from a randomized field experiment. Journal of the Society for Social Work and Research.

[CR82] Metzler CW, Sanders MR, Rusby JC, Crowley RN (2012). Using consumer preference information to increase the reach and impact of media-based parenting interventions in a public health approach to parenting support. Behavior Therapy.

[CR83] Miller WR, Rollnick S (2013). Motivational interviewing: Helping people change.

[CR84] Ministry of Housing, Communities & Local Government. (2021). *Troubled families annual report of 2021. *Retrieved from https://www.gov.uk/government/publications

[CR85] Moore KJ, Patterson GR, O’Donohue WT, Fisher JE (2009). Parent training. General principles and empirically supported techniques of cognitive behavior therapy.

[CR86] Morris AS, Criss MM, Silk JS, Houltberg BJ (2017). The impact of parenting on emotion regulation during childhood and adolescence. Child Development Perspectives.

[CR87] Mytton J, Ingram J, Manns S, Thomas J (2014). Facilitators and barriers to engagement in parenting programs: A qualitative systematic review. Health Education and Behavior.

[CR870] Mytton JA, Ingram J, Manns S, Stevens T (2014). The feasibility of using a parenting programme for the prevention of unintentional home injuries in the under-fives:a cluster randomised controlled trial. Health Technology Assessment.

[CR88] National Child Abuse Statistics from NCA. (n.d.). National Children’s Alliance. Retrieved November 1, 2023, from https://www.nationalchildrensalliance.org/media-room/national-statistics-on-child-abuse/

[CR89] Oliver J, Washington KT (2009). Treating perpetrators of child physical abuse: A review of interventions. Trauma, Violence, and Abuse.

[CR90] Osman F, Vixner L, Flacking R, Klingberg-Allvin M, Schön UK, Salari R (2021). Impact of a culturally tailored parenting programme on the mental health of Somali parents and children living in Sweden: A longitudinal cohort study. British Medical Journal Open.

[CR91] Parra-Cardona JR, Sharp EA, Wampler RS (2008). “Changing for my kid”: Fatherhood experiences of Mexican-origin teen fathers involved in the justice system. Journal of Marital and Family Therapy.

[CR92] Parra-Cardona JR, Wampler RS, Sharp EA (2006). “Wanting to be a good father”: Experiences of adolescent fathers of Mexican descent in a teen fathers program. Journal of Marital and Family Therapy.

[CR93] Patterson GR, Fagot BI (1967). Selective responsiveness to social reinforcers and deviant behavior in children. The Psychological Record.

[CR94] Petersen V, Steinman SB (1994). Helping children succeed after divorce: A court-mandated educational program for divorcing parents. Family and Conciliation Courts Review.

[CR95] Pinto R, Canário C, Leijten P, Rodrigo MJ, Cruz O (2024). Implementation of parenting programs in real-world community settings: A scoping review. Clinical Child and Family Psychology Review.

[CR96] Pollet SL, Lombreglia M (2008). A nationwide survey of mandatory parent education. Family Court Review.

[CR97] Press S (2013). Family court services: A reflection on 50 years of contributions. Family Court Review.

[CR98] Prinz RJ, Sanders MR, Shapiro CJ, Whitaker DJ, Lutzker JR (2009). Population-based prevention of child maltreatment: The U.S. Triple P system population trial. Prevention Science.

[CR99] Prinz RJ, Sanders MR, Shapiro CJ, Whitaker DJ, Lutzker JR (2016). Addendum to “Population-based prevention of child maltreatment: The U.S. Triple P system population trial. Prevention Science.

[CR100] Pritzker, J. B., & Smith, G. M. D. (2021). *Illinois Department of Children & Family Services Family First Prevention Services Act Title IV-E Prevention Plan*.

[CR101] Puzzanchera C, Hockenberry S, Sickmund M (2022). Youth and the juvenile justice system: 2022 National Report.

[CR102] Rimestad ML, Lambek R, Zacher Christiansen H, Hougaard E (2019). Short- and long-term effects of parent training for preschool children with or at risk of ADHD: A systematic review and meta-analysis. Journal of Attention Disorders.

[CR103] Roeder-Esser C (1994). Families in transition: A divorce workshop. Family and Conciliation Courts Review.

[CR104] Rogers-Brown JS, Self-Brown S, Romano E, Weeks E, Thompson WW, Whitaker DJ (2020). Behavior change across implementations of the SafeCare model in real-world settings. Children and Youth Services Review.

[CR105] Romano E, Gallitto E, Firth K, Whitaker D (2020). Does the SafeCare parenting program impact caregiver mental health?. Journal of Child and Family Studies.

[CR106] Rostad WL, McFry EA, Self-Brown S, Damashek A, Whitaker DJ (2017). Reducing safety hazards in the home through the use of an evidence-based parenting program. Journal of Child and Family Studies.

[CR107] Salem P, Sandler I, Wolchik S (2013). Taking stock of parent education in the family courts: Envisioning a public health model. Family Court Review.

[CR108] Sanders MR (2008). Triple P-Positive Parenting Program as a public health approach to strengthening parenting. Journal of Family Psychology.

[CR109] Sanders MR (2012). Development, evaluation, and multinational dissemination of the Triple P-Positive Parenting Program. Annual Review of Clinical Psychology.

[CR110] Sanders M, Calam R, Durand M, Liversidge T, Carmont SA (2008). Does self-directed and web-based support for parents enhance the effects of viewing a reality television series based on the Triple P-Positive Parenting programme?. Journal of Child Psychology and Psychiatry.

[CR111] Sanders MR, Kirby JN (2014). A public-health approach to improving parenting and promoting children’s well-being. Child Development Perspectives.

[CR112] Sanders MR, Kirby JN, Tellegen CL, Day JJ (2014). The Triple P-Positive Parenting Program: A systematic review and meta-analysis of a multi-level system of parenting support. Clinical Psychology Review.

[CR113] Sanders MR, Mazzucchelli TG (2013). The promotion of self-regulation through parenting interventions. Clinical Child and Family Psychology Review.

[CR114] Sanders MR, Mazzucchelli TG (2022). Mechanisms of change in population-based parenting interventions for children and adolescents. Journal of Clinical Child and Adolescent Psychology.

[CR115] Saunders R, Brack M, Renz B, Thomson J, Pilling S (2020). An evaluation of parent training interventions in Scotland: The Psychology of Parenting Project (PoPP). Journal of Child and Family Studies.

[CR116] Schaeffer CM, Swenson CC, Powell JS (2021). Multisystemic Therapy—Building Stronger Families (MST-BSF): Substance misuse, child neglect, and parenting outcomes from an 18-month randomized effectiveness trial. Child Abuse and Neglect.

[CR117] Schaffner L (1997). Families on probation: Court-ordered parenting skills classes for parents of juvenile offenders. Crime and Delinquency.

[CR118] Self-Brown S, McFry E, Montesanti A, Edwards-Gaura A, Lutzker J, Shanley J, Whitaker D, Reece RM, Hanson RF, Sargent J (2014). SafeCare: A prevention and intervention program for child neglect and physical abuse. Treatment of child abuse: Common ground for mental health, medical, and legal practitioners.

[CR119] Sexton T, Turner CW (2010). The effectiveness of functional family therapy for youth with behavioral problems in a community practice setting. Journal of Family Psychology.

[CR120] Shapiro C, Prinz R, Sanders M (2010). Population-based provider engagement in the delivery of evidence-based parenting interventions: Challenges and solutions. Journal of Primary Prevention.

[CR121] Sloan E, Hall K, Moulding R, Bryce S, Mildred H, Staiger PK (2017). Emotion regulation as a transdiagnostic treatment construct across anxiety, depression, substance, eating, and borderline personality disorders: A systematic review. Clinical Psychology Review.

[CR122] Solomon DT, Niec LN, Schoonover CE (2017). The impact of foster parent training on parenting skills and child disruptive behavior: A meta-analysis. Child Maltreatment.

[CR123] Sotero L, Cunha D, da Silva JT, Escudero V, Relvas AP (2017). Building alliances with (in)voluntary clients: A study focused on therapists’ observable behaviors. Family Process.

[CR124] Staudt M, Cherry D (2009). Mental health and substance use problems of parents involved with child welfare: Are services offered and provided?. Psychiatric Services.

[CR125] Stoltenborgh M, Bakermans-Kranenburg MJ, Alink LRA, van IJzendoorn MH (2015). The prevalence of child maltreatment across the globe: Review of a series of meta-analyses. Child Abuse Review.

[CR126] Sumargi A, Sofronoff K, Morawska A (2015). A randomized-controlled trial of the Triple P-Positive Parenting Program seminar series with Indonesian parents. Child Psychiatry and Human Development.

[CR127] Swenson CC, Schaeffer CM, Henggeler SW, Faldowski R, Mayhew AM (2010). Multisystemic therapy for child abuse and neglect: A randomized effectiveness trial. Journal of Family Psychology.

[CR128] Thomson RN, Carlson JS (2017). A pilot study of a self-administered parent training intervention for building preschoolers’ social-emotional competence. Early Childhood Education Journal.

[CR129] Tomlinson CS, Rudd BN, Applegate AG, Holtzworth-Munroe A, Fagan J, Pearson J (2021). Challenges and opportunities for engaging unmarried parents in court-ordered, online parenting programs. New research on parenting programs for low-income fathers.

[CR130] U.S. Department of Education. (2004). *Individuals with Disabilities Improvement Act of 2004, Public Law 445, U.S Statutes at Large 118 (2004)* (pp. 2647–2808).

[CR146] U.S. Department of Health and Human Services. (2016). Head Start performance standards. https://eclkc.ohs.acf.hhs.gov/policy/45-cfr-chap-xiii

[CR132] U.S. Department of Health & Human Services, Administration for Children and Families, Administration on Children, Youth and Families, Children’s Bureau. (2024). Child maltreatment 2022. Retrieved from https://www.acf.hhs.gov/cb/data-research/child-maltreatment

[CR133] Valleley RJ, Meadows TJ, Burt J, Menousek K, Hembree K, Evans J, Gathje R, Kupzyk K, Sevecke JR, Lancaster B (2020). Demonstrating the impact of colocated behavioral health in pediatric primary care. Clinical Practice in Pediatric Psychology.

[CR134] van der Stouwe T, Asscher JJ, Stams GJJM, Deković M, van der Laan PH (2014). The effectiveness of Multisystemic Therapy (MST): A meta-analysis. Clinical Psychology Review.

[CR135] Webster-Stratton C (1985). The effects of father involvement in parent training for conduct problem children. Child Psychology and Psychiatry and Allied Disciplines.

[CR136] Wells KC, Griest DL, Forehand R (1980). The use of a self-control package to enhance temporal generality of a parent training program. Behaviour Research and Therapy.

[CR137] Welmers-van de Poll MJ, Roest JJ, Stouwe T, Akker AL, Stams GJJM, Escudero V, Overbeek GJ, Swart JJW (2018). Alliance and treatment outcome in family-involved treatment for youth problems: A three-level meta-analysis. Clinical Child and Family Psychology Review.

[CR138] Whitaker DJ, Ryan KA, Wild RC, Self-Brown S, Lutzker JR, Shanley JR, Edwards AM, McFry EA, Moseley CN, Hodges AE (2012). Initial implementation indicators from a statewide rollout of SafeCare within a child welfare system. Child Maltreatment.

[CR139] Whitaker DJ, Self-Brown S, Hayat MJ, Osborne MC, Weeks EA, Reidy DE, Lyons M (2020). Effect of the SafeCare© intervention on parenting outcomes among parents in child welfare systems: A cluster randomized trial. Preventive Medicine: An International Journal Devoted to Practice and Theory.

[CR140] Wiggins TL, Sofronoff K, Sanders MR (2009). Pathways Triple P-positive parenting program: Effects on parent-child relationships and child behavior problems. Family Process.

[CR141] Windell JO, Windell EA (1977). Parent group training programs in juvenile courts: A national survey. The Family Coordinator.

[CR142] Wolchik SA, Sandler IN, Winslow EB, Porter MM, Tein J (2022). Effects of an asynchronous, fully web-based parenting-after-divorce program to reduce interparental conflict, increase quality of parenting and reduce children’s post-divorce behavior problems. Family Court Review.

[CR143] Wolford SN, McWey LM (2020). “It makes me step back a little…check myself”: Parental identity and mandated participation among parents involved with the child welfare system. Journal of Family Violence.

[CR144] Yamaguchi R, Randel B (2021). Evaluation of an online parenting and divorce course: Effects of parent knowledge and skills among court-mandated parents of divorce. Journal of Divorce and Remarriage.

[CR145] Zhou YQ, Chew QRC, Lee M, Zhou J, Chong D, Quah SH, Ho M, Tan LJ (2017). Evaluation of Positive Parenting Programme (Triple P) in Singapore: Improving parenting practices and preventing risks for recurrence of maltreatment. Children and Youth Services Review.

